# Current concepts in multiparametric magnetic resonance imaging for active surveillance of prostate cancer

**DOI:** 10.6061/clinics/2018/e464s

**Published:** 2018-11-27

**Authors:** Alexandre Cavalcante, Públio Cesar C Viana, Giuliano B Guglielmetti, José Pontes Junior, Henrique Nonemacher, Mauricio D Cordeiro, Regis Otaviano F Bezerra, Rafael F Coelho, William Carlos Nahas

**Affiliations:** I Instituto do Cancer do Estado de Sao Paulo (ICESP), Hospital das Clinicas HCFMUSP, Faculdade de Medicina, Universidade de Sao Paulo, Sao Paulo, SP, BR; II Grupo de Uro-Oncologia, Departamento de Urologia, Faculdade de Medicina FMUSP, Universidade de Sao Paulo, Sao Paulo, SP, BR

Active surveillance (AS) is a possible new approach for men with very low-risk and low-risk prostate cancer (PCa) and, less frequently, those with intermediate-risk PCa as a way to reduce overtreatment of clinically indolent disease 1-3. The very low likelihood of progression of this disease to unfavorable outcomes in appropriately selected men has demonstrated that AS can be used with good outcomes 4,5. High cancer-specific survival rates have been described, reaching 94.3% and 99.9% at the University of Toronto and Johns Hopkins Hospital, respectively 4,5, during 15 years of follow-up. Data from Sweden and the United States have shown cancer-specific survival rates of 60% and 64%, respectively, in patients who participated in AS after 5 years 6,7, and 55% cancer-specific survival has been reported in Canada after 15 years 4.

Despite good outcomes with AS, the poorly defined criteria for intervention and the lack of standardized surveillance protocols are important limitations. In all protocols for AS around the world, systematic transrectal ultrasound (TRUS)-guided biopsy is the tool used to establish a diagnosis of PCa 8, but the morbidity associated with this method is well known and can range from local symptoms, such as hematuria and acute urinary retention, to systemic symptoms, such as bacteremia and sepsis. Each new biopsy greatly increases the complication rates 9.

Another issues related to AS is the underestimation of the real extent of the disease and aggressiveness based only on standard biopsy (SB) data. Approximately thirty percent of patients with low-risk PCa on initial biopsy have a Gleason score upgrade when treated by radical prostatectomy; thus, SB is associated with substantial misclassification that may lead to inappropriate selection of patients for AS and postponement of the necessary treatment 10. Therefore, distinction of patients who have low-risk cancers and those who need immediate treatment is a serious challenge. In this scenario, multiparametric magnetic resonance imaging (mpMRI) has appeared as a promising imaging modality to help to better select and manage patient candidates for AS 11-13.

Currently, many studies confirmed that mpMRI is a useful instrument for detecting clinically significant PCa. The sensitivity is 86% for identifying tumors greater than 0.5 cm^3^ in size 14 and 80% for detecting index tumors 15. However, the precise role of mpMRI in AS is not yet well established. Defining the role of mpMRI during the initial diagnosis and follow-up of patients undergoing AS is the next step.

## mpMRI

In recent years, rapid technological advances have taken place. Thus, the acquisition of high quality images has advanced the current knowledge about the findings of PCa on mpMRI. Exam standardization and a high level of reader training have made mpMRI an important tool in PCa management in daily practice. Usually, the exam is performed by a 3-Tesla scanner; however, a 1.5-Tesla magnet with an endorectal coil can be used to perform diffusion weighted imaging (DWI), T2-weighted imaging (T2WI) and dynamic contrast-enhanced MRI (DCE-MRI) 16.

The famous Prostate Imaging Reporting and Data System version 2 (PI-RADS), which is frequently used in the description of PCa, was developed to standardize MRI reporting. PI-RADS can be used to generate a map of the prostate to define the area of interest 17. Suspicious lesions are graded on a 5-point scale based on features suggestive of malignancy, and the probability of clinically significant PCa is related to a higher PI-RADS grade. The lesion with highest PI-RADS category if there is more than one lesion or the largest lesion when the lesions have same PI-RADS grade is considered the index lesion.

**Figure 1:**
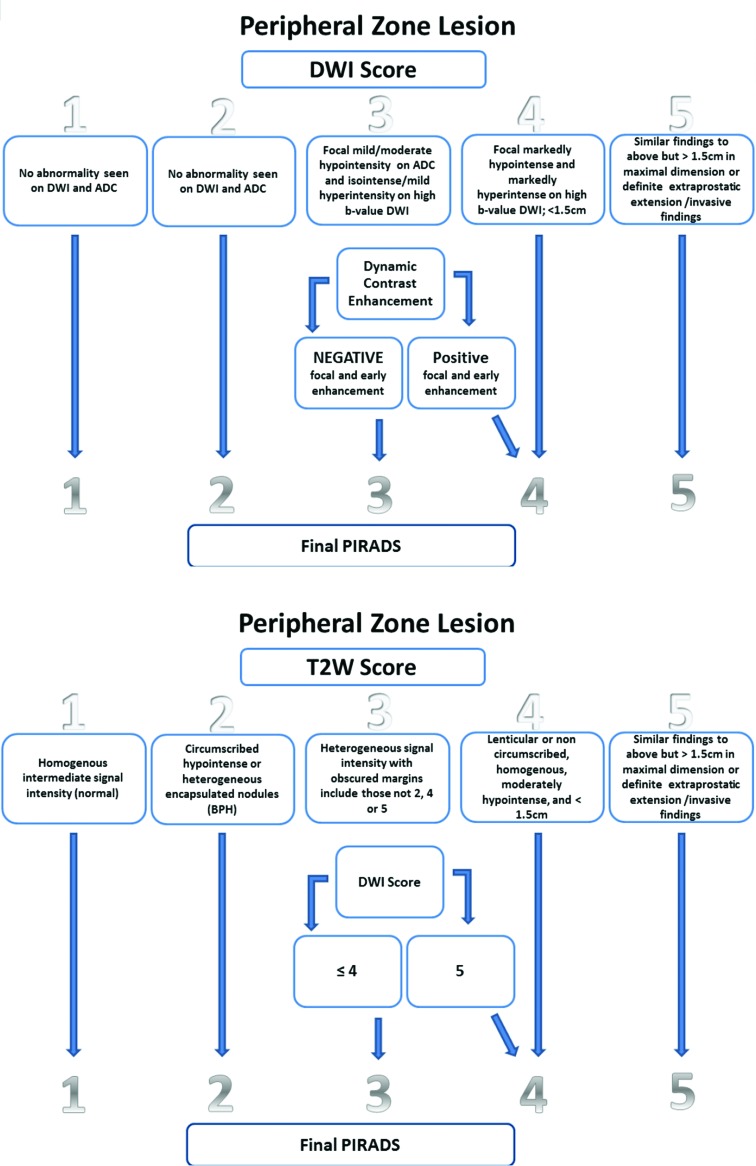
DWI is better than T2WI in the peripheral zone, and T2WI is better than DWI in the transitional zone. The DCE-MRI determines the final PI-RADS score when PI-RADS 3 is present in the peripheral zone. DWI determines the final PI-RADS score when PI-RADS 3 is present in the transitional zone.

Certain characteristics of the parameters of T2WI, DWI and DCE-MRI aid in disease diagnosis. Currently, the use of DCE-MRI has low applicability. DCE-MRI is used only for lesions in in situ irradiated glands in which an enhancing nodule is strongly suggestive of PCa or to separate PI-RADS 3 and 4 lesions in the peripheral zone. Greer et al. demonstrated a high positive predictive value for PI-RADS ≥3 and ≥4 lesions (85% and 90%, respectively) to detect clinically significant cancer with PI-RADS 2.0 18 (Figure 1).

## Candidate selection for AS and the role of mpMRI

There are several AS protocols described in the literature with different inclusion criteria, and the lack of uniformity among these protocols poses the first challenge in clinical practice (Table 1). The most frequently used criteria to include patients in AS are Gleason score ≤3+3, prostate-specific antigen (PSA) ≤10 ng/mL, clinical stage ≤T2, maximum of 2 positives cores or ≤50% per core. Two important exceptions are the groups from Memorial Sloan-Kettering Cancer Center who admitted patients with up to three involved cores and the group from Toronto who admitted patients with Gleason scores up to 7 (3+4) and with PSA levels up to 15 ng/mL. When the patients do not fulfill all these criteria, they cannot be included in AS because are deemed to have clinically significant disease. The problem with AS is that conventional prostate biopsy undersamples roughly one-third of patients when compared with prostatectomy specimens 20. mpMRI can yield important details about the disease since this modality detects with higher accurately tumors with Gleason score higher than 6 and has a higher sensitivity than computed tomography for detecting extracapsular extension thus decreasing the undergrading and understaging rates. However, in the majority of AS programs, mpMRI findings are not adopted as initial inclusion criteria.

Currently, mpMRI has helped in the early detection and staging of PCa. Many studies have demonstrated that the incorporation of mpMRI findings during the initial diagnosis of PCa has better accuracy than TRUS findings 21,22. If MRI shows a suspected lesion, these patients must undergo image-guided fusion biopsies. There are three possible modalities that can be used to perform this procedure: in-bore MR fusion, cognitive fusion, and finally, MR-US software fusion biopsy 16. Overall cancer detection is significantly higher when MR-US software is used to generate the fusion image (48.1%) than when cognitive fusion alone is used (34.6%) (*p*=0.04) 23.

Siddiqui et al. 24 recruited 1003 men with PCa who underwent SB and targeted biopsy. This prospective study showed that targeted biopsy had increased accuracy for high-risk cancer, diagnosing 30% more cases (173 *vs.* 122 cases, *p*<0.001) and decreased accuracy for low-risk cancer, diagnosing 17 fewer cases (213 *vs.* 258 cases, *p*<0.001). In a second analysis evaluating targeted biopsy combined with SB, an increase in the diagnosis of low-risk PCa with an additional 103 cases (22%) was observed. Among 170 patients who underwent radical prostatectomy and were diagnosed with whole-gland disease after pathologic analysis, the preoperative targeted biopsy demonstrated better predictive ability to differentiate low-risk, intermediate or high-risk tumors than the two approaches used together or SB alone (0.73, 0.67 and 0.59, respectively, *p*<0.05) 24.

Pessoa et al. 25 recently published a prospective cohort study involving 105 patients on AS who underwent mpMRI examinations. Reclassification rates among patients with PI-RADS 1, 2, 3, 4, and 5 were 0%, 23.1%, 9.1%, 74.5%, and 100%, respectively. The overall reclassification rate was 55.2%, and the most frequent criterion change was Gleason score classification (44.8%). Approximately 60% of the population was PI-RADS 4 and 5 on mpMRI, and in this group (PI-RADS grade 4 and 5), the rate of reclassification was 85.71%. The sensitivity, specificity, positive predictive value, and negative predictive value of mpMRI for disease reclassification were 92.5%, 76%, 81%, and 90.5%, respectively. These findings are comparable to what Lee et al. 26 found at the Cleveland Clinic on prostate specimens from patients who underwent radical prostatectomy for low-risk disease.

Other similar studies have been recently published. Da Rosa et al. 27 from the University of Toronto, in a prospective study, reported upgrading in 19 out of 72 patients on AS with a Gleason score ≥7. Seven of these upgraded patients were detected only on MR-fusion-targeted biopsy, two were detected only on TRUS-guided systematic biopsy alone, and 10 were detected using both techniques. The negative predictive value for significant cancer approached 100% among men with no suspicious lesions identified using mpMRI. Current data from Memorial Sloan-Kettering 28 have been reported regarding the reclassification rate of 206 men with low-risk PCa enrolled in AS who underwent MR-fusion-targeted biopsy and systematic prostate biopsy. MRI identified suspicious lesions in 66% of patients, reinforcing that MRI findings have the potential to alter the management of low-risk PCa patients.

In a retrospective study, Vargas et al. 13 found that 20% (79 of 388) of patients with clinically low-risk PCa had Gleason scores upgraded on confirmatory biopsy. These patients underwent MRI scans between the initial and confirmatory biopsies. MRI scores ≤2 had a high negative predictive value (0.96-1.0) for upgrading on confirmatory biopsy, while a score of 5 were highly sensitive for upgrading on confirmatory biopsy (0.87-0.98).

In summary, the use of mpMRI as initial screening exam for PCa allows better stratification of the disease. The sensitivity and predictive negative value of mpMRI are high, both of which are important features for screening tests. MRI fusion biopsy of target lesions has higher predictive negative than TRUS-guided biopsy 29.

## Role of mpMRI during follow-up of patients on AS

Once patients are selected to undergo AS, they are monitored with PSA exams, digital rectal exams (DREs) and standard prostate biopsies. Sometimes patients are followed for many years and may require a considerable number of biopsies. However, prostate biopsy is an invasive technique and is associated with morbidity. The most common complications are pain, hematuria, hematospermia, erectile dysfunction, and infections. Thus, some recent studies have demonstrated that mpMRI is a promising alternative to decrease biopsies and thus prevent the complications related to this method.

The true role of mpMRI for monitoring patients on AS is not fully established; however, some studies suggest that serial MRI scans improve prediction of pathological progression compared to clinicopathological variables alone, and stable MRI findings are associated with pathological stability 30-32. In the largest published retrospective study, Frye et al. 33 analyzed 166 men with PCa on AS in whom mpMRI-evident lesions were monitored, and fusion-guided biopsy was properly indicated. The mean follow-up was 25.5 months, and pathologic progression was observed in 29.5% of patients. Targeted fusion biopsy identified progression in 44.9% of patients, and systematic biopsy detected progression in 30.6% (*p*=0.03). Progression was observed 26% more frequently with fusion biopsy than with systematic biopsy, and in this series, progression on mpMRI was shown to be the sole predictor of pathological progression in patients on AS (*p*=0.013). The analysis of pathologic progression on mpMRI in a cohort study demonstrated a negative predictive value of 81%, a sensitivity of 77.6%, a positive predictive value of 35% and a specificity of 40.5%. These authors concluded that as imaging and technology evolve, the number or frequency of biopsies in men on AS may potentially be reduced 33.

In their cohort, Felker et al. 30, found that mpMRI had low sensitivity (37%) but high specificity (90%) for predicting pathologic progression. The main changes were maximum cancer core length (MCCL) ≥3 mm at baseline biopsy and prostate-specific antigen density (PSAD) ≥0.15 ng/ml at follow-up biopsy. Overall, 19 patients had pathologic progression: 9 patients (47%) were identified on targeted biopsy, 7 (37%) on systematic biopsy, and 3 (16%) on systematic and targeted biopsy, highlighting the importance of targeted biopsies in patients on AS. In a similar study, Rosenkrantz et al. 32 analyzed and compared the changes in prostate index lesions identified on serial mpMRI with the follow-up biopsy results of patients on AS. A total of 55 patients were analyzed with median follow-up of 14 months; these authors observed that MRI had a high specificity (76% to 90%) for predicting positive follow-up biopsy results but a low sensitivity (23% to 35%).

In 206 men selected for AS, Recabal et al. 28 noted that 66% had regions of interest on MRI, and 35% were upgraded. They also found that when MRI-targeted biopsy was performed, the increase in detection of higher grade cancer was 23%; furthermore, the higher the PI-RADS score on MRI was, the greater the likelihood of finding a high-grade tumor (*p*<0.0001).

Morgan et al. 34 published a pilot study with 50 patients on AS who underwent mpMRI and compared the differences in apparent diffusion coefficients (ADCs) in patients who underwent radical prostatectomy and those who did not. A 10% decrease in ADC designated progression with a sensitivity of 93%. Thus, this study demonstrated that MRI may be an effective tool for follow-up of patients with early PCa on AS.

Tran et al. 35 evaluated the utility of mpMRI fusion biopsy for predicting disease progression in patients on AS compared with systematic biopsy. They showed that 83 men (40%) experienced upgrading including 49 (24%) who underwent systematic sampling, 30 (14%) who underwent MRI-targeted core biopsies, and 4 (2%) who underwent both. Seven patients (9%) exhibited major upgrading with systematic biopsy among those with negative results on MRI-US fusion biopsy.

## Cost-effectiveness analysis of mpMRI during AS

An important point for deciding whether a method should be used routinely in the investigation and follow-up of a disease is whether this method has clinical applicability and feasibility from an economic point of view. However, this analysis becomes difficult since the cost of a specific exam and its particularities, such as hospitalization, treatment of complications and protocols for institutional execution of the exams, are extremely variable around the world. A few studies have attempted to analyze the cost-effectiveness of mpMRI in patients on AS and the results have varied. The methods and parameters used to evaluate cost-effectiveness in these studies were not uniform; thus, making comparisons of the results is not possible. The main points evaluated in the cost-effectiveness analysis studies were the estimated cost of the examination and the quality of life assessed by quality-adjusted life years (QALYs). QUALYs is a generic measure used in economic evaluation to assess the value for money of medical interventions; one QUALY is equal to to one year in perfect health and 0 QUALY indicates death.

European studies have had divergent results about the cost-effectiveness of MRI for AS 36-38. In these studies, the costs of MRI and TRUS were similar. Nicholson et al. 36 showed that the use of MRI to follow-up patients with PCa on AS was not cost-effective when compared with other strategies. Two other studies demonstrated divergent results, stating that MRI-based strategies were cost-effective 37,38. Mowatt et al. 38 suggested that the use of MRI in the patients studied was £7,685 below the threshold stipulated by QUALY (£20,000 *vs* £12,315) calculated in order to be considered cost-effective in the UK. In a study conducted in the Netherlands, de Rooij et al. 37 also showed the cost-effectiveness of strategies based on MRI.

In an Australian study, Gordon et al. 39 analyzed the survival, total number of biopsies, healthcare costs, insignificant and significant cancer and QUALYs in patients in 3 different scenarios: patients diagnosed by TRUS without MRI and referred for radiotherapy, surgery or AS; patients who underwent TRUS guided by MRI and referred for radiotherapy, surgery or AS; and patients who underwent TRUS guided by MRI who all underwent AS. After separately analyzing the cost of the exam and QUALY, it was not possible to state that an MRI was cost-effective in patients with PCa in the current scenario of AS. However, it is believed that if MRI is able to include more patients on AS thus postponing radical treatment (surgery or radiotherapy), this tool can become cost-effective. A recent study entitled the ProstateMR Imaging Study (PROMIS) was conducted to analyze the cost of diagnosing clinically significant (CS) cancer by comparing MRI with TRUS findings in the UK 40. The results showed that MRI as the first examination detected more CS cancer per pound than TRUS (sensitivity =0.95 [95% confidence interval (CI) 0.92-0.98] *vs* 0.91 [95% CI 0.86-0.94]) and was also cost-effective (ICER=£ 7,076 [€8350 / QALY gained]). Even though the PROMIS study did not aim to evaluate cost-effectiveness in AS patients, we can infer that this study has indirect applicability in the AS scenario, since diagnosing patients with CS cancer earlier can lead to exclusion or inclusion of these patients in AS programs.

Due to the wide divergence between studies in the analysis of the cost of MRI compared to TRUS or the cost-effectiveness of these techniques used during AS, we cannot extrapolate the results of one study to a location other than where the study was conducted. In addition to cost variability among exams, hospitalizations and procedures worldwide, the lack of uniformity in the protocols used for AS make it difficult to standardize cost-effectiveness studies. Thus, at this time, it is not possible to make final statements about the cost-effectiveness of MRI in the AS scenario. Individualized studies for the given reality in which the use of mpMRI is desired to be evaluated seem to be the best option for a more reliable analysis of cost-effectiveness.

Current data show that mpMRI is an adjunctive powerful tool that can assist in the selection of patients for AS, increasing the diagnostic accuracy of prostate biopsy. The integration of mpMRI data and the combination of targeted biopsy and systematic biopsy improve detection of higher grade disease thus decreasing incorrect patient selection for AS. Therefore, we believe that MRI findings should be used as additional criteria for inclusion of patients in future protocols for AS. The role of MRI in the follow-up of patients on AS is not yet well established. Recent data support that MRI should not be used as an autonomous tool to follow-up and trigger biopsies because upgrading also occur in areas outside the targeted biopsy, but improved definition and standardization of radiological progression for patients on AS is expected.

## Figures and Tables

**Table 1:** Active surveillance protocols. Inclusion criteria, monitoring and intervention criteria.

Cohort	Selection criteria	Monitoring protocol	When to intervene
Johns Hopkins Tosoian et al. (19) 2015	Gleason ≤ 3 + 3 T1c ≤2 positive cores ≤50% involvement in any corePSA density <0.15 ng/mL	DRE and PSA measurements (total and free) every 6 months Annual 12- to 14-core biopsy	Gleason >6 >2 positive cores >50% involvement in any core
Sunnybrook (Toronto) Klotz et al. 2015	Low risk Gleason 3 + 3 PSA <10 ng/mL or Favorable intermediate risk: PSA 10-20 ng/mL Gleason ≤3 + 4	3-monthly PSA for 2 years, then 6-monthly Confirmatory biopsy within 12 months of initial biopsy, then every 3 to 4 years until age 80	PSA doubling time <3 years Upgrade on repeat biopsy Clinical progression on DRE
UCSF Welty et al. 2015	PSA <10 ng/mL T1 or T2 Gleason 3 + 3 or less <33% positive cores <50% cancer involvement in any core	PSA every 3 months TRUS every 6 months Annual 12-core sextant biopsies	Patient anxiety Biopsy reclassification Change in clinical stage CAPRA risk reclassification
Australian Thompson et al. 2014	PSA <10 ng/mL Stage <T2b on DRE Gleason 3 + 3 <20% positive cores <30% or 6 mm cancer in all positive cores	PSA every 3 months for 3 years then 6-monthly DRE every 6 months for 3 years then annually Biopsies at 12- 24-36 months, then every 3-5 years Watchful waiting >75 years or life expectancy <7 years	PSA doubling time <3 years PSA velocity >0.75 ng/mL DRE progression Biopsy upgrade Cancer volume progression
PRIAS Bokhorst et al. 2016	≤Gleason 3+3 ≤T2c PSA <10 ng/ml ≤2 positive cores PSA density <0.2 ng/ml/cm^2^or Gleason ≤ 3 + 4: < 10% involvement in any core, ≤ 2 positive cores if age > 70 or >2 cores positive on MRI-target biopsy 15% involvement in core on saturation biopsies	3-monthly PSA and 6-monthly DRE for the first 2 years 6-monthly PSA and annual DRE thereafter Standard biopsies at 1, 4, 7, and 10 years, then every 5 years Bone scan if PSA >20 ng/ml	>2 positive cores Gleason > 3 + 3 Stage >cT2 PSA doubling time <3 years Criteria adapted for Gleason 3 + 4 and >2 cores based on MRI or saturation biopsies
Royal Marsden Selvadurai et al. 2013	Gleason 3 + 3 Gleason 3 + 4 in patients > 65 PSA <15 ng/ml T1 or T2 PPC <50% of total number of biopsy cores	DRE and PSA every 3 months in the first year Every 4 months in the second year Every 6 months thereafter TRUS biopsy after 18-24 months and then every 2 years	PSAV >1 ng/ml per year Upgrade on repeat biopsy
